# Value-based tiered pricing for universal health coverage: an idea worth revisiting

**DOI:** 10.12688/gatesopenres.13110.3

**Published:** 2020-04-24

**Authors:** Kalipso Chalkidou, Karl Claxton, Rachel Silverman, Prashant Yadav

**Affiliations:** 1 Global Health Policy, Center for Global Development, London, UK; 2 Medicine, School of Public Health, Imperial College London, London, UK; 3 Department of Economics, University of York, UK, York, UK; 4 Technology and Operations Management, INSEAD, Fontainebleau, France

**Keywords:** UHC, Universal Health Coverage, Tiered Pricing, Value-based

## Abstract

The pricing of medicines and health products ranks among the most hotly debated topics in health policy, generating controversy in richer and poorer markets alike. Creating the right pricing structure for pharmaceuticals and other healthcare products is particularly important for low- and middle-income countries, where pharmaceuticals account for a significant portion of total health expenditure; high medicine prices therefore threaten the feasibility and sustainability of nascent schemes for universal health coverage (UHC). We argue that a strategic system of value-based tiered pricing (VBTP), wherein each country would pay a price for each health product commensurate with the local value it provides, could improve access, enhance efficiency, and empower countries to negotiate with product manufacturers. This paper attempts to further understanding on the potential value of tiered pricing, barriers to its implementation, and potential strategies to overcome those.

## Background

The pricing of medicines and health products has become one of the most hotly debated topics in health policy—in both industrialized/OECD countries and low- and middle-income countries (LMICs). Creating the right pricing structure for pharmaceuticals and health products has become particularly important for LMICs, where pharmaceuticals account for a significant portion of total health expenditure.

Under the historical model for global health, most of the world’s poor lived in low-income countries; the global burden of disease was largely communicable, and mostly concentrated in low- and lower-middle-income countries; and development assistance for health financed large portions of health expenditure in low and lower-middle income countries. This model of global health assistance is now passé. The world’s poor (and disease burden) are no longer concentrated in low-income countries (LICs) but now reside primarily in middle-income countries (MICs). And as MICs transition from health assistance, they often face significantly higher prices for health products compared to the prices received by global health mechanisms. This poses a significant risk to sustaining the health gains achieved in immunization, HIV/AIDS, tuberculosis (TB), malaria, family planning, and other disease areas funded though development assistance for health. 

At the same time, other forces are at work. Epidemiological transition is shifting the disease burden from infectious to non-communicable diseases (NCDs) across most countries, but many LMICs still face a high communicable disease burden—especially in Sub-Saharan Africa and South Asia. Economic growth and improved fiscal space have not necessarily translated into commensurate increases in government health spending ( Doherty *et al.*, 2018; Tandon *et al.,* 2018). The modest increases in government expenditure for health are insufficient to sustain financing of health programs previously supported by donors, particularly given that countries face higher prices of health products. As donor aid shrinks, and government expenditures do not increase fast enough to keep pace, a majority of the health expenditure in LMICs (especially for medicines/health products) is borne out-of-pocket by individuals and families. For some transitioning countries, high prices for vaccines, antiretroviral therapy (ART) and other products can jeopardize the financial sustainability of health sector budget ( Silverman, 2018). When faced with the choice between sustaining donor-financed programs and making other investments in the health system or NCDs, some country governments are inclined to choose the latter—leading to tensions between different global agencies and country governments that further complicate this issue.

The lack of a clear architecture for pricing and prioritization of health products continues to be a major impediment to achieving UHC ( Schäferhoff *et al.*, 2019; Watkins *et al.*, 2018). National health insurance systems—most of which are still in stages of infancy in most LMICs—are devoting large portions of their limited budgets to health products (e.g. Ghana’s NHIS and Kenya’s NHIF spend between 40–55% of their total budgets on health products); this makes it difficult to expand quality services to cover more of the population given most developed systems devote a much smaller fraction of their budgets on commodities versus salaries and infrastructure. In tandem, new technologies (especially vaccines and diagnostics) are entering LMIC health systems at an unprecedented pace. The pressure to adopt new health technologies creates significant opportunity costs and is likely to crowd out or prevent investment in other more cost effective interventions, though wat exactly gets displaced is much harder to assess as opposed to populations and things that are simply not covered (e.g. Kenya and GeneXpert ( Callaway, 2017; Muchangi, 2019); Senegal offering free access to trastuzumab, which has in turn been shown not be cost-effective in most African settings ^1^ ( Gershon *et al.*, 2019); or the recent listings in the WHO Essential Medicines List of expensive products for cancer and autoimmune diseases, including erlotinib and adalimumab, with incremental cost effectiveness ratios in the order of hundreds of thousands of dollars, in the hope generic versions will materialise ( Hill *et al.*, 2016; WHO, 2019—page 222); or dialysis in LMICs absorbing large chunks of small and strained budgets ( van der Tol *et al.*, 2019), whilst countries such as Kenya are foregoing financing essential products such as family planning commodities which are currently wholly financed through development assistance monies).

This context requires rethinking the current model of pharmaceutical pricing—and, we argue, an important role for value-based tiered pricing (VBTP), a system of pricing where each country pays a price commensurate with local value. In this article we first describe the review the theoretical and empirical literature on the potential benefits and challenges of applying tiered pricing schemes in LMICs. We then present VBTP as an approach to help manage some of the tensions and trade-offs in the debate around LMIC medicine pricing. While VBTP is no magic bullet for universal LMIC access to medicines, it holds the potential to move countries forward toward UHC.

## Theory and evidence on pricing models and differential pricing

### Overview

Efficient pricing for on-patent pharmaceuticals is complex and challenging. Under the current IP model, private companies pay upfront for pharmaceutical R&D (though they often benefit from public sector investments in basic scientific research and early stage R&D); they later recoup their upfront investments and earn profits by selling successful pharmaceutical innovations at prices well above marginal cost, protected by term-limited patents ( Institute of Medicine (US) Forum on Drug Discovery, Development, and Translation, 2009). If prices are too low, pharmaceutical companies will not invest in innovation (dynamic inefficiency); if prices are too high, the costs of accessing existing therapies will outweigh the benefits (static inefficiency). Pricing policy for innovative pharmaceutical therefore need to achieve a delicate balance between these twin goals: affordable access to existing treatments on the one hand, and potentially transformative and lifesaving innovation on the other.

Zooming out from pricing policy in any individual country, global pricing for on-patent products becomes even more challenging. Pharmaceutical R&D is a global public good; the cost is borne through a one-time upfront investment justified by the potential for future sales, and the benefits are potentially shared across consumers worldwide. Prices are not determined by the costs of R&D; however, the size of investment in R&D is determined by the expectations of the global prices manufacturers are likely to command for the products in development. Since post-hoc sales indirectly fund R&D in this way, the choice of pricing policy across countries therefore determines how much each country contributes to the joint cost of pharmaceutical innovation. (For products that are off-patent, market competition has the potential to create the right pricing structure—though in practice, market failures and barriers to entry often help sustain artificially high prices in LMICs even after patent expiration ( Silverman, 2018).

For products under patent and with limited therapeutic substitutes, price is determined by strategic responses between the manufacturer and purchasers in different markets. The manufacturer’s goal is to maximize total profits across all markets. Each purchaser, in contrast, aims to access the drug at the lowest possible price, so long as the benefits at that price outweigh the cost of the drug. (For simplicity, the below section refers interchangeably to “countries” and “consumers”, imagining that each country is served by a single payer able to negotiate and purchase on behalf of all citizens using pooled funds; in practice, however, most markets are served by a heterogenous mix of public payers, private insurers, and out-of-pocket expenditure from individuals.)

One possibility would be for the manufacturer to set a uniform single price for all countries across the entire world; countries could choose to either purchase the drug at the uniform price or walk away without purchasing the drug. At a very high price, the manufacturer knows that only a few countries will be willing to pay for the drug and total revenue will be low. At a very low price, on the other hand, almost every country will be willing to pay for the drug—but total revenue will still be low because revenue per-pill will be miniscule and may even fall below the marginal cost of production. The optimal price, from the perspective of the patent holder, would be somewhere in the middle, where the marginal revenue of serving one additional country—accounting for both increased volume, which increases revenue; but also decreased price across *all* countries, which decreases revenue—is equal to the marginal cost of producing the pill. That is, the manufacturer will choose the single price for the entire world based on the overall/aggregate demand elasticity.

But a single price would create significant social dead-weight loss (inefficiency). Some countries would be unwilling or unable to buy the drug at the single uniform price; that means some markets would not be served at all ( Kremer & Snyder, 2018). A uniform price would also be suboptimal from the manufacturer’s perspective, since it leaves potential revenues from unserved markets on the table; therefore, a single price would also be inefficient for recouping R&D costs and incentivizing future innovation ( Danzon, 1997).

Alternatively, a manufacturer could deploy differential pricing across multiple heterogenous markets—that is, a manufacturer could charge different prices for the same product in different countries. Price differences would reflect differences in the willingness (and ability) of each country to pay for the product. (More specifically the manufacturer would charge lower prices to price-sensitive countries, and higher prices to less price-sensitive countries).

In theory, differential pricing across countries can create welfare gains by improving access for patients in developing countries without necessarily harming either the profits of the pharmaceutical companies or access for patients in developed countries. Under certain conditions, differential pricing may also lead to better incentives for pharmaceutical research and development, and thus in the long run could benefit patients in both developing and developed countries ( Danzon & Towse, 2003).

### Literature review

Yadav (2010) provides a review of literature on differential pricing. Relevant literature is reviewed below to provide the right theoretical background for the rest of this paper.

***Price discrimination in a monopoly.*** Multiple studies ( Schmalensee, 1981) have shown that differential pricing by a single profit-maximizing manufacturer leads to improvements in overall welfare (i.e. benefits both the manufacturer and the consumers) if total sales increase as a result of differential pricing. Similar findings are reported in Varian (1985) and Schwartz (1990). Layson (1994) shows that if a monopolistic firm serves two markets—one with higher willingness/ability to pay and larger profit margin, and a second with lower willingness/ability to pay but a large market size—price discrimination will enhance social welfare. More generally, Malueg & Schwartz (1994) show that price discrimination increases social welfare when there are large differences in demand. Hausman & Mackie-Mason (1988) note that price discrimination is also more likely to increase dynamic welfare by better incentivizing research and development.

***Price discrimination in an oligopoly.*** There is very little research that examines the impact of price discrimination in an oligopoly (products with a small number of manufacturers, but more than one). Using a simplified model, Fudenberg & Tirole (2000) predict that price discrimination among firms in an oligopoly would lead to high initial prices followed by a subsequent price reduction; consumers would be better off in aggregate.

***When one market is a monopoly and other market is oligopoly.*** In some cases, a firm could serve two independent markets—one in which it enjoys a monopoly, and the other in which it must compete with a rival firm. The effect of price discrimination in this scenario remains understudied, though this analysis would be important for understanding situations where generic competition exists in some countries, but other countries remain under patent exclusivity. In this environment, Armstrong & Vickers (1993) show that a firm would set a monopoly price in the first market; prices in the second market would be determined by the competitive interaction between the two firms. If the firm cannot successfully set different prices in the two markets due to price regulation or other factors, the firm may end up choosing a reduced price in the captive market and a raised price in the competitive market.

Under a system of differential pharmaceutical pricing, Mujumdar & Pal (2005) show that price regulation in one county has no impact on prices charged in a second country. As long as there is no price referencing, pricing decisions are made independently for each market.

***Ramsey pricing.*** While in theory differential pricing is based on price elasticity of customer segments, in practice price elasticities of individual customer segments/markets are unobservable. As a result, average per capita income is often used as a proxy for price elasticity and differential pricing is designed around GNI/capita or GDP/capita. While voluntary differential pricing by a manufacturer achieves higher social welfare compared to charging a single uniform price, the absolute price levels charged by a profit-maximizing monopolist may not be socially optimal. Ramsey pricing ( Ramsey, 1927) can be utilized to determine differential prices in each market to recoup the variable and joint R&D costs. Ramsey pricing, which was originally explored as a pricing approach for public utilities with large fixed costs, involves choosing prices in each market in inverse relation to the demand elasticities, and subject to assuring a specified target profit level for the manufacturer, e.g. a firm’s target internal hurdle rate of return. Depending on who sets the target rate of return, Ramsey pricing can be more akin to a regulated profit maximizing monopolist. Even though Ramsey pricing can be welfare efficient, it requires a social planner to set the prices based on an acceptable rate of return for the manufacturer. Without clarity on who sets the prices and what is an acceptable rate of return for the manufacturer, Ramsey pricing may not balance the goals of improving access to LMICs and incentivizing the manufacturer R&D. Without consensus on what would be the socially acceptable rate of return for the manufacturer, the selected price differentials often transfer a larger portion of surplus to the manufacturer, making it similar to a price discriminating monopolist.

***Welfare effects in global markets using simulation or empirical data.*** Several papers use simulation or empirical data to interrogate the welfare effects of price discrimination across global pharmaceutical markets. Dumoulin (2001) uses a simulation model to compare a single global price with differential prices based on country income. The analysis shows that differential pricing maximizes both manufacturer profit and affordability to the population, increasing access by a factor of roughly 4–7. Among countries with the same GDP per capita, the country in which wealth is most concentrated will face a higher price under price discrimination; companies would rationally price for the rich segment of society rather than the more populous but less lucrative lower-income segment.

Scherer (2004) considers the welfare effects of allowing poor countries to access generic versions of medicines protected by patents in rich countries. Globally, he finds this would increase welfare because the marginal utility of income (the benefit derived from one extra unit of currency) is greater in poor nations than in rich ones. However, this may lead to negative welfare effect in the rich countries.

Danzon (1997) compares the welfare effects of differential pricing for pharmaceuticals in the United States and the European Union (EU). They show that prices in the European Union (EU), are farther from ‘Ramsey Optimal Prices’ due to parallel trade and monopsony buying structure. Danzon & Chao (2000) compared the prices of a limited sample of drugs across countries and conclude that prices for generics are lower in markets without price regulation.

Hellerstein *et al.*, (2004) examine differential pricing for ARVs for HIV/AIDS and show that until 2000 there was little variation in the prices of ARVs between the high and low-income countries.

Reich & Bery (2005) discuss differential pricing among various options of improve access to AIDS medicines. They list three possible mechanisms for a differential pricing system: internal company polic- based differential pricing, international agency facilitated differential pricing and wider distribution of price information to different actors.

Lopert *et al.* (2002) recommend a mechanism of setting prices in each country based on the incremental cost per life-year gained for each country based on its per capita gross domestic product (GDP) as a proxy for a patient’s ability to pay.

Based on an extensive literature search, Lang & Hill (2004) conclude that differential pricing can lead to improved access for low-income countries, increased market share for companies, and no price increases for high-income countries.

***Summary.*** Compared to a single price across countries, the literature clearly demonstrates that differential pricing enhances both static and dynamic efficiency. The debate, therefore, is not about the value of differential pricing *per se* but rather how differential pricing should be structured; how it should distribute surplus between the manufacturer and buyer(s); and, relatedly, how it should trade off between dynamic and static efficiency. Traditional price discrimination allows the manufacturer to capture a larger portion of the economic surplus, therefore privileging R&D investment (dynamic efficiency); Ramsey-style pricing, in contrast, could in theory transfer most of the surplus to consumers and regulate profits, therefore privileging affordable access to existing therapies (static efficiency).

### Challenges in conventional differential pricing

The theoretical benefits of differential pricing can only be met under specific conditions. Manufacturers must be able to securely separate economic markets based on demand elasticity—preventing either physical or informational leakage between markets and using the right proxies for demand elasticity in each.

In LMICs, these conditions are rarely met in practice ( Yadav, 2010). Many LMICs have highly skewed income distributions, making it more lucrative to cater to richer segments of society than the more voluminous poorer classes (oftentimes including large migrant/undocumented populations from neighbouring countries). This is exacerbated by relatively low levels of pooling; most purchase decisions, in practice, are made by individuals, who are likely to be more price sensitive than a pooled payer. Manufacturers are also wary of physical arbitrage (e.g. reimportation to higher value markets) and informational arbitrage (e.g. lower LMIC prices used to inform pricing in higher value markets via external reference pricing). Drug resistance and supply/manufacturing constraints may also create impediments for serving more price-sensitive consumers ( Yadav, 2010). In addition, while overall welfare may be higher, tiered pricing may allow producers to charge a margin which is far higher than the amount needed to recoup R&D investments and production costs ( Moon *et al.*, 2011).

Manufacturers face additional demand uncertainty when serving low and middle income markets ( Levine *et al.*, 2008). Such uncertainty prohibits them from better production planning and other measures which can bring production efficiency.

Further, in LMIC markets, driven perhaps by the lack of a central negotiator in the form of a National Health Insurance Fund or national purchasing agency on behalf of the healthcare system and the presence of significant income inequalities, there have been recent examples of industry-led attempts to price discriminate within a country. AstraZeneca’s Mosaic Segmentation approach for antihypertensive medication in Brazil, Takeda’s partnership with Axios to assess patient financial eligibility for its expensive Hodgkin’s lymphoma and irritable bowel syndrome medication in the Philippines, Thailand and central Asia and Novartis’ Potential Affordability by Decile accounting for income levels to assess affordable for out of pocket patients are examples cited as best practices by the 2018 and 2019 Access to Medicines Index reports and analyses, 2018. Whilst an laudable effort in the face of weak and fragmented demand side in many LMICs, industry driven VBP based on very rough subgroup segmentation based on income levels is not sustainable (with every company devising a scheme for select products), nor can it account for the budgetary constraint (other than through rough estimates of individual income and abilities to pay). Most importantly, our proposal hinges on the presence of pooled financing and purchasing at the country (or at least regional in countries with devolved federal structures) level where a dedicated purchasing function with the capacities to assess value given budget size and potential alternative uses of the latter, and to negotiate, based on this assessment with suppliers, exists.

To achieve welfare enhancing prices, some argue that the monopoly pricing power of a manufacturer must be constrained by regulatory price controls or through competition (e.g. via compulsory licensing). This may achieve static efficiency gains, but the resulting effect on dynamic efficiency could be problematic.

See Table 1 below for a description of the three main product characteristics to consider for the applicability of differential pricing.

**Table 1.** Product and market characteristics for applicability of differential pricing.

Differential pricing can only create welfare gains for certain product and market types. There are three main product characteristics to consider for the applicability of differential pricing:
** 1. Product life cycle:**
• Established products with dozens of generic manufacturers do not require differential pricing; the forces of market competition are the best lever to achieve optimal prices. • New health technologies which are currently being launched in HICs may be a good candidate for differential pricing, allowing simultaneous (versus delayed) market launch in LMICs. • On-patent products that have already been launched in LMICs through donor-led procurement—e.g. new ARVs or vaccines—could also be good candidates for differential pricing
** 2. Production cost and economies of scale:**
• Production of biologics and vaccines requires significant capital investments to set up manufacturing plants; their production cost curves are steep downward sloping. • This implies that differential pricing can enhance economies of scale for one (or a few) manufacturers, allowing lower prices at the lowest tier. • In the long run, however, differential pricing (and the subsequent higher prices) could create barriers to entry/ competition; new manufacturers may not be able to achieve the economies of scale enjoyed by the one or two large incumbent manufacturers.
** 3. Complexity in administering the product:**
• As discussed earlier, the welfare-enhancing properties of differential pricing only apply when differential pricing leads to higher overall sales for manufacturers—that is, when lower prices lead to a higher sales volume. For products that require more sophisticated health system infrastructure to administer, a lower price may not necessarily lead to a substantial increase in sales volume. Differential pricing may not be the best approach for such products unless health system infrastructure is improved in parallel.

### Differential pricing and procurement architecture

Real-world transactions, including manufacturer price-setting, are often more complex than basic economic theory would predict. When a single manufacturer sells to multiple markets, the manufacturer considers not just price elasticity of the market but also the purchaser’s buying power, as well as other factors such as payment timeliness, long-term customer value, and transaction costs. Smaller country purchasers are then at a natural disadvantage; the volume they are purchasing is too small to create negotiating leverage based on market power, and the transaction costs are high relative to a manufacturers’ total potential revenue. In addition, a substantial portion of health procurement is not done at the national level, but instead by smaller purchasers within subnational governments, the private sector, or individual hospitals/facilities, among others.

One option used to increase small countries’(and other purchasers’) buying power and reduce transaction costs is pooled or joint procurement. Pooled procurement has been deployed, for example, via the Gulf Cooperation Council (GCC) pooled procurement mechanism, the Pan-American Health Organization (PAHO) Revolving Fund for Vaccines, the Organisation of Eastern Caribbean States (OECS), and the African Island States procurement service, both for single source/on-patent drugs and generics. Such arrangements can only work if participating countries have comparable income levels and/or willingness to pay, since a single price is set across participating countries.

Monopsony power of large buyers carries some risks and can lead to unintended consequences. For example, if pooled monopsonist purchasers with high income heterogeneity between participating countries use their buying power to exercise lowest price clauses in their price negotiations, it can lead to decreases in welfare ( Privett & Yadav, 2012). A large pooled purchaser which has a combination of low-, middle- and high-income countries in its pool can leverage its buying power to demand low prices for all its members, including middle- and high-income countries. Strategic pricing response from manufacturer to the presence of such a buyer reduced overall welfare in the system. 

## Value-based tiered pricing

In economic theory, true differential pricing should reflect each market’s willingness to pay. In contrast, conventional differential (tiered) pricing used in global health (e.g. for vaccines, ARVs, malaria medicines, and contraceptives ( Yadav, 2010) has primarily used per capita income as a proxy for willingness to pay. The use of GDP per capita as a crude proxy for willingness to pay is a significant flaw in the design of differential pricing programs, driving contention and debate. The highly skewed income distribution in countries such as Brazil, India, and Thailand leads to discontent with prices offered based on national average GNI/capita ( Yadav, 2010).

**Figure 1.**
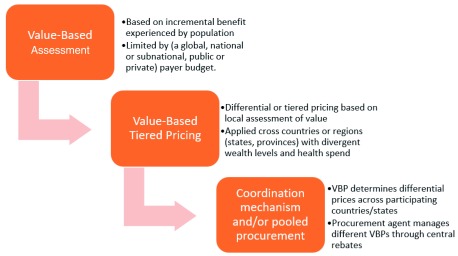
Sequence of actions for value-based tiered pricing.

A different approach—value-based (benefit-based) tiered pricing (VBTP)—has the potential to address previously observed challenges with conventional differential pricing ( Danzon *et al.*, 2015). Under VBTP, prices in each country should be based on a health system’s willingness to pay, where willingness to pay reflects the actual, assessed value of the product within that market/health system (‘value-based pricing’) accounting for affordability and budgetary constraints (See Figure 1) ( Claxton, 2007). The assessed value of a product is based on three factors. First, how much additional health will the product create (compared to the current standard of care or potential comparator products)? Second, what are the net additional costs to the health care system of adoption, including how may decrease or increase health spending elsewhere? (For example, a vaccine would prevent disease, which a health system would otherwise need to pay to treat; but a new medical device would also require implantation or surgical costs in addition to the procurement price of the product.) Finally, how much is the health system willing and able to pay for additional health benefits offered (e.g. per disability- or quality-adjusted life years)?

### Determining value…locally

Use of value assessment to inform pricing decisions is already supported by a substantial research and institutional infrastructure. Many LMICs are already using or exploring value assessment (through health technology assessment) to inform their procurement/price negotiation and reimbursement decisions, including Thailand, China, India, Ghana, South Africa, the Philippines, Indonesia, Brazil Mexico, Colombia, Kenya and Tanzania ( Government launches health technology assessment to inform policy decision making Nairobi, (n.d.); Hollingworth *et al.,* 2019; MacQuilkan *et al.,* 2018; Surgey *et al.,* 2019; Tantivess *et al.,* 2017). There is also a large body of literature around how to estimate a health system’s willingness and ability to pay for health gains.

The key question is what improvement in health would be possible if the additional (net) resources required had, instead, been made available for other health care activities. This assessment of health opportunity cost is relevant whether the additional costs of the investment must be found from existing commitments and current levels of health expenditure, or when health expenditure can/will be increased to accommodate the additional resources required. Therefore, the problem of establishing how much a health care system should be willing and able to pay for the benefits of a product is the same as estimating the relationship between changes in health care expenditure and health outcomes. Countries vary in disease burden, demographics, health expenditure and system structure. As a consequence the marginal productivity of health care expenditure, health opportunity costs, and how much health care systems can afford to pay for the health benefits of products are likely to be correlated with income levels; however, GDP per capita, by itself, will not precisely predict optimal pricing.

Estimates of the marginal productivity of health expenditure in producing health (quality-adjusted life-years) are becoming available for some high-income countries based on approaches to estimation which exploit within-country data, ( Claxton *et al.*, 2015; Edney *et al.*, 2018; Lomas *et al.*, 2018; Martin *et al.*, 2008; Vallejo-Torres *et al.,* 2018; for a critique see). This evidence from high-income settings can be used to give some indication of possible values in lower income countries ( Woods *et al.*, 2016) based on a number of assumptions about income elasticity of demand for health and the relative ‘under funding’ of health care systems.

The effect of different levels of health care expenditure on mortality outcomes has been investigated in a number of published studies using country level data, many including LMICs ( Gallet & Doucouliagos, 2017). The challenge is to control for all the other reasons why mortality might differ between countries in order to isolate the causal effect of differences in health expenditure. A number of studies try and overcome this problem and estimate outcome elasticities for all cause adult and child mortality, by gender, as well as survival, disability and DALYs ( Bokhari *et al.,* 2007; Ochalek *et al.*, 2018). These estimated elasticities have been used to provide country specific estimates of health opportunity costs (cost per DALY averted) for 97 LMICs, taking account of measures of a country’s infrastructure, donor funding, population distribution, mortality rates, conditional life expectancies (all by age and gender), estimates of disability burden of disease and total health care expenditure ( Haasis *et al.*, 2015). These types of estimates suggest that optimal prices under VBTP will vary across countries and as a general trend increase with increasing GDP per capita, but specific pricing in each country would depend on how much health would be produced in that system, the specific cost structure of the health system, and how a country values health gains achieved through a health technology. 

VBTP therefore offers an opportunity to improve upon conventional tiered pricing and sustainably capture the potential efficiency gains from differential pricing, particularly in countries which are moving towards high coverage in their national insurance programs and are already using value-based pricing and cost-effectiveness thresholds. Value assessment provides a more accurate measure of a country’s willingness to pay than use of GDP per capita, which is likely to be correlated with willingness to pay but offers only a crude, often imprecise proxy. Definitionally, VBTP also ensures that a product would be locally affordable, as the value assessment explicitly considers a country’s willingness to pay for health gain, which is directly linked to its ability ** to pay for health gain, given resource constraints. 

### “What’s in it for me?” Benefits of VBTP for different actors in the health system

Below we describe how a functional VBTP system would benefit different actors within LMIC health systems. As described in the previous section, our definition of “value” is always based on benefits that accrue to the payer and the payer’s willingness to pay for these benefits.

***Health care systems in low- and middle-income markets.*** A VBTP system would dramatically increase the accessibility and affordability of health innovation in LMICs. VBTP would ensure that transaction prices are affordable, reflect local opportunity costs, and therefore remove the politically difficult choice between restricting access to effective medicines or allowing access at too high a price, thereby damaging other parts of the health care system and the economy. By using the benefits-based price as the starting point/ceiling price in negotiations with industry, country payers can ease budget pressure, making collective or universal coverage more politically and financially sustainable. In particular, VBTP can help relieve budget pressure within the MICs currently setting up insurance or universal health coverage schemes and payer agencies, especially when existing health technology assessment infrastructure is already driving listing decisions and price negotiations. Using a VBP as the starting point for further negotiations, is not inconsistent with the notion of rewarding a product’s value, in that both development partner funds in the case of LMICs and national government monies in the case of HICs make major contributions to infrastructure, professional education and directly towards early stage high risk research all of which are of the essence in successful privately funded R&D. Given there is limited empirical evidence of how the surplus ought to be shared between demand and supply side during monopoly protection, negotiations based on evidence of value seem to us to be a good compromise.

In the absence of tiered pricing (e.g. at a single global price), most MICs would be priced out of the market for innovative pharmaceuticals, except perhaps among a handful of wealthy individuals willing and able to pay out of pocket. In contrast, prices that reflect the budgetary constraint can help drive appropriate and equitable uptake of branded pharmaceuticals, provided arrangements are in place to adjust the value-based price downwards ^2^ in situations where adoption would lead to significant budget impacts (e.g. introduction of PCV in the Philippines ( Ochalek *et al.*, 2018); or HepC drugs in Australia). Appropriate VBTP implies that a country payer can afford to purchase an innovative product for the entire ** covered population in need—avoiding inequities caused by rationing either on an *ad hoc* basis or based on individuals’ ability to pay out-of-pocket. Likewise, differential pricing across countries implies that a greater number of countries could afford health innovation—reducing cross-country inequities of access and health outcomes (though better outcomes for many NCDs such as cancer or diabetes also require significant expenditure and capacity elsewhere in the health system, e.g. cancer outcomes depend on early diagnosis, access to radiotherapy, and surgery etc.).

Likewise, a global system of VBTP would help signal LMIC health needs and demand to product developers, creating at least some influence on commercial research and development decisions (to a significant extent compared to single global price ^3^). Private sources invest over $170 billion each year in healthcare R&D; over time, consistent demand signaling from LMICs should direct at least some of these resources toward LMICs’ specific health needs. The strength of this signal will grow smoothly and progressively with economic growth rather than the ‘binary’ effect of a single global price. Further, as MICs invest in their own healthcare product industries, a VBTP system could expand the size of domestic and global pharmaceutical markets. The Chinese government, for example, is already emphasizing R&D (including biosimilars and increasingly “innovative” products) within the Chinese pharmaceutical industry with the government emphasis on R&D including biosimilars.

Importantly, such an R&D system is still likely to underserve low-income countries (LICs), which are shrinking in number and population but continue to represent a substantial portion of the global disease burden. LICs are likely to have different innovation needs than MICs (e.g. treatment/prevention for neglected tropical diseases or products that can be delivered without cold chains), extremely limited ability to pay for innovation, and continued reliance on donor funding and procurement support. However, a VBTP could still benefit LICs by improving access to existing products with a shared burden across LICs and MICs/high-income countries (HICs)—so long as the LIC value-based price still exceeds the manufacturers’ marginal cost of production.

More broadly, in situations where the value- or benefit-based price an LMIC is less than the manufacturer’s marginal cost of production, the manufacturer will still have no commercial incentive to offer the product for sale, even under a system of perfect global price discrimination. This scenario may be quite common in LICs, where evidence-based, budget-sensitive thresholds are likely to be much lower than those used by global partners (or formerly by WHO) and even small molecules may be too costly at marginal-cost prices ( Chalkidou & Keller, 2017). Further, the production costs of new biologics (and biosimilars) remain very high also requiring significant upfront investment costs in manufacturing plants (unlike small molecule generics). In addition, the total cost of serving an additional market also includes the costs associated with regulatory approval, product launch, and safe delivery in the context of care pathways and with associated systems—which may be cost-ineffective, given competing priorities and limited budgets, in many LICs and some MICs. So, while a VBTP system should improve access to some portion of innovative health products in at least some markets, perfect price discrimination alone will not, unfortunately, lead to full access for all branded drugs in all contexts.

***Industry.*** VBDP would increase industry’s revenue and profit for a product during patent protection. Pricing (and volumes) in each country would be based on evidence of a product’s value proposition—a rational and therefore accountable assessment for each health system. A VBTP system would be more predictable than current arrangements, where regulatory approval is disconnected from listing decisions and listing decisions themselves offer little information on the likely extent of take by the health system. As such, price discrimination reduces unnecessary risks while maintaining companies’ incentives to develop better products, as more effective drugs will predictably increase revenue while an ineffective or dangerous product would yield no revenue at all.

It is theoretically possible that perfect VBTP across systems would lead to lower revenue than the status quo for some products. If this is the case, however, this implies that some countries are presently overpaying ** for pharmaceuticals relative to the opportunity cost; this means current revenues are likely to be unsustainable in the medium-term regardless as they would be unsustainable for the healthcare system. Indeed, VBTP is by design sustainable, as healthcare payers will be able, definitionally, to afford access at price offered in their setting. This may help reduce the likelihood that national payers adopt more drastic policy responses to address unaffordable pharmaceutical prices, which may include:

a.   Arbitrary regulatory, evidential, and budgetary barriers to entry which prevent market access and increase development costs

b.   Competitive tendering or other all-or-nothing aggressive price negotiation tactics by monopsonists.

c.   Complete abandonment of patents as products are unaffordable (e.g. through compulsory licensing). 

The clear demand signals from MICs (see previous section) would also create additional commercial opportunities for innovator companies to serve MIC health needs, widening the pipeline of products and disease areas as the healthcare industry gradually shifts focus to fast-growing emerging markets.

***Global agencies and development partners.***

• Better targeting of non-commercial R&D investment

In the context of the aid transition, there is growing concern that available resources for assistance are being spent on wealthier countries as opposed to the poorest ones in need (e.g. see latest analysis on EU aid ( Morton *et al.*, 2018)). Aid should not be used to purchase products at unnecessarily high prices in situations where price discriminate could enhance access to needed medicines at locally affordable prices. Development partner subsidies should instead be devoted to the poorest countries, who cannot afford basic health products even at marginal cost, and real sources of market failure where global public goods are needed, for example R&D targeting the poorest nations. While continued aid will still be needed to fund R&D when a market’s commercial proposition remains unattractive, VBTP would make some R&D investment commercially viable; commercial viability would also increase so with expectations of economic growth across LMICs. In turn, agencies, donors and foundations could focus their investments where there is insufficient commercial incentive for market forces to be effective.

• More efficient development partner commodity portfolio

Similarly, publicly funded conduits, such as the Global Fund to Fight AIDS, Tuberculosis, and Malaria (Global Fund), can use VBTP calculations to guide their own investment portfolios ( Grubert, 2019; Isabelle *et al.*, 2017). With more than half of the Global Fund’s $14 billion budget going to commodities (many of which remain on-patent), the lack of a mechanism for *ex ante* value assessment risks compromising value-for-money VFM and setting unaffordable pricing precedents price that cannot be sustained following aid transition ^4^. Similarly, other global players such as CHAI and UNITAID can use value-based assessments to negotiate market shaping deals, (i.e. for dolutegravir) and advise on post-transition listing/procurement decisions.

• Value-informed market shaping

Where donors still play a role in market shaping, VBTP can address the current lack of consideration for products’ comparative clinical value or global/national affordability. Initiatives such as MedAccess, which helps manage the risk of new product launches in LMICs, can use VBPT to anchor price negotiations with manufacturers, helping ensure better value for the HIC taxpayers which financially support MedAccess and helping signal appropriate country-by-country price points for co-financing.

• Affordable and predictable LMIC government co-financing during aid transition

A VBTP system could be used in tandem with aid, especially in the context of aid transition. Development partners are imposing increasing requirements LMICs to take on part or all of the cost for select products. VBTP can help inform decisions about the appropriate price point for country co-financing of donor-funded products; for example, LMICs could be asked to pay up to the locally-affordable value-based price, while donors pay the remainder of the purchase price ( Claxton, 2007; Claxton *et al.*, 2011).

• Pooled purchasing arrangements for specific product types

VBTP, with each country paying prices commensurate with local value, can form the basis of multinational, cross-country pooled purchasing agreements for certain types of products suffering from specific market failures, including unpredictable demand or onerous country-by-country launch requirements (e.g. cancer drugs, biosimilars, and insulin products). Such arrangements already work in HIC settings, where countries are forming buyer coalitions for horizon scanning, joint price negotiations, and procurement deals ( PMPRB, 2019). Such arrangements could be organized by LMICs themselves or with support from development partners.

In summary, VBTP can improve the value-for-money of health aid, benefiting both HIC taxpayers and aid beneficiaries, including LMIC governments.

### Pre-requisites for VBTP

For a global VBTP to work in practice, several preconditions must be in place—some of which are not yet fully operational in many LMICs:

•   A functional regulatory system trusted by consumers and professionals;

•   Purchasing/reimbursement and price negotiation arrangements through a national or subnational payer able signal willingness to pay based on budgetary constraints; and

•   A functional, financed through pooled funding, healthcare system through which the population can consistently access procured health products.

In addition, VBTP would be most effective when complemented by the following conditions, which would allow for healthy market competition:

•   A competitive, quality-assured generics market, resulting in significant price reductions in following patent expiry;

•   Acceptance of the current patent system for most products (with possible exceptions for antimicrobials, and other specific cases) and with modest modifications to prevent abuses observed under the status quo (e.g. to remove practices such as evergreening).

### Potential challenges

***Countries with large out-of-pocket markets.*** The VBTP approach would work well in countries where a single or handful or purchaser/payers make reimbursement decisions for the entire population. But despite progress, most LMICS still lack insurance coverage for a majority of the population; most still purchase medicines out of pocket. The demand curves for individual patients’ out-of-pocket demand are very different from population level willingness to pay, making it harder to implement any form of VBTP, especially as the poorest and sickest individuals tend to be the most price sensitive.

***External reference pricing.*** One major obstacle to a global VBTP system is the risk posed by external reference pricing, where some countries or payers benchmark the prices that they are willing to pay to the prices paid by other countries or payers. Such a system may achieve short-term price reductions for individual payers, but in the long run external reference pricing leads prices to converge towards a single global price—likely close to the current high prices observed in the United States due to the and pull of the American market (see theory section for further discussion of this phenomenon).

To address this challenge, any individual healthcare system could adopt a value-based pricing and price negotiation mechanism with confidential rebates—some of which are already in use. The United Kingdom’s National Institute for Health and Care Excellence (NICE), for example, negotiates confidential rebates with manufacturers to maintain a distinction between the observed price paid and the true net transaction price for the NHS. However, since confidential discounts are applied to each individual technology NICE assesses, it is still technically possible (though hardly straightforward) to identify the transaction price for each product, and for that price then to be referenced in other systems. Though this is rarely or never done in practice under the current international pricing system, expansion of VBTP to other large markets might create additional incentive to uncover the confidential prices achieved by other payers.

As an alternative to avoid some of these issues, one could envisage a two-way value-based rebate. This system would include a minimum volume/revenue guarantee offered to the manufacturer, and a maximum cost guarantee offered to the payer at the national (or international, in the case of a cross country pooled procurement mechanism) level. This would make it harder to identify transaction prices for specific products. The English NHS has applied this mechanism (where English regions could emulate national or subnational payers participating in an international value-based pooled procurement approach) ( Nemzoff *et al.,* 2019); see also Canada and its provinces where VBP has recently become law ( Syam, 2014) further discussed here ( Glennie, 2019).

***Free riding and other challenges.*** As long as some countries remain outside a VBTP system, there remain incentives for some purchasers to free ride by selectively referencing lower prices from other systems. Importantly, this is not just a problem for VBTP, but applies to any pricing model besides a uniform single price.

One way of addressing this challenge would be an international commitment to prevent parallel trade and/or external reference pricing, potentially administered and enforced through the WTP. However, arriving at such an agreement would be politically difficult; as just one example, the protection of parallel trade between EU states is enshrined in EU law). Even if this initial barrier could be overcome, further challenges would arise related to monitoring and enforcement; agreeing on and implementing a mechanism to calculate value-based prices in each healthcare system (e.g. capacity and technical constraints or gaming); and ensuring that real transaction prices remain difficult to detect.

Although the difficulties are considerable, the potential gains are large. And despite national and EU legislation, pooled procurement and joint price negotiation (with different prices per country) are already taking place through country coalitions such as BeNeLuXa for select products. Further, the current political appetite in the US to reduce prices can make VBTP—which would likely result in lower than current price in the US—a politically viable option.

***Global price transparency:*** Whilst far from endorsed let alone implemented (or indeed implementable) across member states, WHA 2019 called for transparency of costs of R&D and of prices across all products and all countries. A single global price or at least significant price convergence, which are most likely to result from price transparency (unless countries formally agree not to reference price one another and to pay higher prices than their neighbours if they are wealthier) cannot, by definition, reflect value locally and may well delay or complete impede access to the extend manufacturers will opt for wealthier larger markets such as the US vs poorer European ones which may be in turn reference priced by US payers as recently called for by President Trump and the Democrats. We discuss the implications of price transparency especially for governments and individuals in LMICs, elsewhere ( Chalkidou & Towse, 2019).

***What if a global agreement is not possible? Starting with NCDs or products affected by aid transition?*** An alternative starting point would include LMICs and development partners with a particular focus on one or more specific diseases (e.g. NCDs such as cancer and diabetes, or TB/malaria/HIV), products (e.g. vaccines, family planning commodities), or populations (e.g. displaced populations/refugees). Both countries and development partners have experimented with pooling procurement (e.g. see ( Jack, 2019) and references therein for an overview; and ( Wilkinson *et al.*, 2016) for the EAC experience). However, neither donors nor national governments in LMICs have seriously considered combining such pooling together with some form of differential pricing.

In the case of development partners, the Global Fund and Gavi have periodically considered price discrimination but abandoned it, in part due to technical and informational challenges and in part due to political pressure from disease and access to medicines advocates who fiercely (and correctly, in our view) oppose a model of value-based pricing dictated by the healthcare products industry ^5^. The Global Fund’s Pooled Procurement Mechanism, StopTB’s Global Drug Facility, and UNICEF’s Supply Division are examples of pooled procurement—but they too tend to secure a single price across the countries on behalf of whom they buy, sometimes as a deliberate policy decision (though in some cases only specific countries, e.g. Gavi-eligible or transitioning countries, may be eligible for specific UNICEF vaccine prices). Market shaping efforts and volume guarantees do not consider incremental benefit-related value (other than price minimization through higher volumes) from the perspective of the purchaser or the end beneficiary. Instead the emphasis has traditionally been on volume guarantees ( Lomas *et al.*, 2018) and underwriting the risk of shortfalls or delays in payment.

Countries have also tended to join forces for achieving a single price, with PAHO’s revolving fund for vaccines and the more recent strategic fund for NCDs and Hep C being cases in point. There again the fund insists on a single price across all participating countries. A similar approach for NCD products through PAHO’s strategic fund has proven less successful.

We posit that insistence on a single price, delinked from payers’ incremental value, is no longer sustainable. Instead, in the context of aid transition, expanding UHC, and growth of emerging markets’ purchasing model, pooled procurement coupled with VBTP becomes a viable proposition.

In this case, a single product-specific purchaser representing a block of country payers would commit to purchasing the appropriate total volume at the average value-based (benefit-based) price—that is, the weighted average of VBPs across participating payers). For manufacturers, this would be equivalent to a situation of perfect price discrimination across the participating countries (or states/provinces in the case of large and diverse federal countries such as India and China). Each payer would then purchase at their particular value-based price from the single global purchaser. Existing mechanisms of underwriting payment risk, especially for poorer purchasers, could still apply as needed; however, country purchasers would play a major role in product selection and in assessing local value.

Where do we start? No such single purchaser exists, but the Global Fund already purchases on behalf of most low- and middle-income countries for specific products. An endorsement of Health Technology Assessment by the routinely carried out by HICs and successful MIC health systems such Thailand and China, would allow it to assess value for each product across participating countries, ideally using accepted standards such as the iDSI Reference case, to inform country-specific prices ( Lomas *et al.*, 2018). Gavi could also play a similar role. This approach would benefit transitioning countries while also improving donors’ value for money and rationalizing co-financing requirements for beneficiary countries. 

Of course, the Global Fund, Gavi, and other specific donor institutions only cover limited disease areas. One might envision a similar approach for cancer drugs (e.g. biosimilars or biologics), autoimmune conditions, or diabetes products. Such pooling and VBTP would only work for products that are not commoditized (generics); in the latter case, different arrangements to increase the competitiveness of generics markets would be needed and VBTP would add little value.

## A research and action agenda

What we propose is not an easy solution but a necessary step to progress the discussions regarding innovation and access to products in emerging economies. One of our reviewers highlights a series of challenges in making VBTP a reality.

Further research is required to scope out the viability of a global VBTP mechanism, its welfare impact and its distributional consequences. The future research agenda might include themes such as an analysis and evaluation of possible mechanisms and institutions; or applying game theory to understand the dynamic interactions between different actors in the healthcare system and consequent welfare effects. Important research questions include:

- What are the benefits and drawbacks of alternative mechanisms/institutional options?
- What is scale of potential value that different stakeholders could achieve through a functional VBTP mechanism? How large is this value relative to total current revenue or current health? Such an assessment would inform the appropriate effort (in time, resources, and political capital) that could be spent to achieve a workable VBTP process.
- Linked to the above, how important are the pre-requisites for achieving VBTP listed earlier (e.g. functional regulation, a national purchaser etc) in facilitating (or impeding) implementation of VBDP in LMICs and to what extent application of VBDP to select products through an HTA mechanism could help countries meet these pre-requisites?
- How motivated would different players be to participate in this system? What could be done to better motivate players who would have less to gain?

Additional empirical research will be needed to address questions central to making a VBTP mechanism work, such as:

- Shortlist of appropriate products for applying the VBTP mechanism and appropriate payers to join.
- Assessment of evidence-informed prices and volumes.
- Assessment of health effects and system costs across the range of population subgroups that could benefit from a selected product (drawing on the iDSI Reference Case).
- Designing/agreeing/establishing an accountable process for coming to scientific value judgments about what the evidence and analysis suggests (drawing on the work of iDSI, DCP and others as well as national payers such as NICE, NIPH and HITAP).
- An estimate of thresholds relevant to each healthcare system, ideally distinguishing the effects of changes in public and private spend (plus their interaction with GDP per capita, drawing on previous work).
- How best to consider how the surplus ought to be shared between manufacturer and buyer during a product’s monopoly period, including how one considers early stage high risk public investment in science and infrastructure and the interplay between pricing and other important policy tools such as patent characteristics, tax environment and industrial policy. The question of how to share the surplus is essentially an empirical one (other than it is not less than zero and no more than 100%) and is effectively about the value of the additional future innovations are by offering a greater share. The answer to this combined with patent length and a discount rate, would allow one to back solve for the dynamic price (which may be greater or less than health opportunity costs). This empirical question is very much under investigated. As things stand, patents are a crude way to try to achieve dynamic prices. Conditional on believing that current levels of patent protection are ‘optimal’ (bar ever-greening and other rent seeking practices) then paying at health opportunity cost is (by assumption) the dynamic price and by implication the resulting share is ‘optimal’.

## Data availability

No data are associated with this article.
