# Supplementary material for: Value-based tiered pricing for universal health coverage: an idea worth revisiting
Source: Gates Open Res. 2020 Apr 24;4:16. Originally published 2020 Feb 4. [Version 3] doi: 10.12688/gatesopenres.13110.3 (PMC7059551; doi:10.12688/gatesopenres.13110.3)
Supplement: Peer review history

# Peer review: Reviewer response for version 3

**DOI:** 10.21956/gatesopenres.14310.r28791

**Published:** 2020-04-27

**Authors:** Hector E. Castro

**Affiliations:** 1 Pharmaceutical Economics & Financing, Management Sciences for Health (MSH), Arlington, VA, USA

Very interesting paper, all suggestions were addressed. The authors added references as requested, explained/qualified various statements regarding potential challenges in making VBTP a reality.

Does the article adequately reference differing views and opinions?

Partly

Are all factual statements correct, and are statements and arguments made adequately supported by citations?

Partly

Is the Open Letter written in accessible language?

Yes

Where applicable, are recommendations and next steps explained clearly for others to follow?

Partly

Is the rationale for the Open Letter provided in sufficient detail?

Yes

Reviewer Expertise:

Pharma-economics and financing

I confirm that I have read this submission and believe that I have an appropriate level of expertise to confirm that it is of an acceptable scientific standard.

# Peer review: Reviewer response for version 2

**DOI:** 10.21956/gatesopenres.14289.r28584

**Published:** 2020-03-05

**Authors:** Lyudmila Nepomnyashchiy

**Affiliations:** 1 Community Health Acceleration Partnership (CHAP), New York City, NY, USA

Summary of the article: This article provides a case for why the global health community should embrace value-based tiered pricing as an approach to incentivizing innovation sustainably while optimizing access to life-saving, cost-effective products. The article provides a literature review on the value of differential pricing and focuses significantly on what could be done specific to LMICs, including the roles that donors, partners and countries could play. The article also reviews specific challenges and potential solutions that could be explored.

 Specific suggestions:

- In Background, paragraph 2: It might be good to more explicitly call out how MICs will have reduced eligibility for development assistance for health. It would be good to prove that this is true (we know that 36/73 GAVI countries will transition away from GAVI assistance by 2030, not sure what the plan is for GF). 
- Background, paragraph 4: It's not clear why 40-55% of budgets spent on health products doesn't cover the population sufficiently. If you were able to contrast that with the percentage of the population that can access NHIS, then it would demonstrate what I think you want to say which is that they spend too much money on commodities while covering a limited percentage of the population. The example of vaccines entering LMICs isn't that applicable yet because Kenya & Ghana are GAVI countries for some time. 
- In the same paragraph, the explanation on how new technologies put pressure on decision-making and raise the stakes to assess opportunity cost is really helpful. However, it's not clear what is cost-effective that is being crowded out. The rest are examples of new technologies that are requiring a lot more money from countries but the opportunity cost isn't clear (perhaps a few examples of those would be helpful much like the stream of examples of the pricey commodities).

Theory and evidence on pricing models & differential pricing - Overview:

- Paragraphs 4 & 5 might benefit from a graph that shows various scenarios & implications (in particular the deadweight loss from a single price) - readers who don't have an economic background might get confused otherwise.

Challenges in conventional differential pricing: 

- Another point that may be relevant to highlight in this section might be related to a few other factors that drive up high prices in LMICs, namely: volatile demand (a supplier can't start and stop production, there are fixed costs and lots of inefficiencies without visibility on capacity needs; prices may be high to compensate for uncertainty with demand). Suppliers' limited visibility on demand in LMICs limits their ability to schedule production, procurement and deliveries. The solution to this becomes standardized, centralized and longer-term procurement (mentioned later in the procurement architecture section). Additionally, any special regulatory approvals or packaging also adds to cost (common with LMICs).
- In research and action agenda - I think it would be helpful to dig a bit into what it would take to get to the pre-requisites mentioned previously (e.g., many LICs are way off from functional regulatory systems!) and to have some idea of how those pre-requisites compare in terms of impact (high/medium low) and feasibility within LMIC settings where these pre-requisites are weak. An argument could be made that VTBP would strengthen all of that - and starting with 1-2 products could sow the seeds for this on a wider scale (e.g., like HITAP?).
- The reason why I said 'partly' for different views & opinions is because this is mentioned in the end as a suggestion for further research (alternative mechanisms/institutional options). Why should LMICs embrace VBTP vs. other pricing policies? How should pharmaceutical policy evolve in these challenging circumstances? I suspect that is enough for another paper and this one should focus but just wanted to call this out. Perhaps it is possible to reference alternatives earlier on (besides just one global price which is described well theoretically but not practical). 

Does the article adequately reference differing views and opinions?

Partly

Are all factual statements correct, and are statements and arguments made adequately supported by citations?

Yes

Is the Open Letter written in accessible language?

Yes

Where applicable, are recommendations and next steps explained clearly for others to follow?

Yes

Is the rationale for the Open Letter provided in sufficient detail?

Yes

Reviewer Expertise:

pricing policies in LMICs, optimizing medicine access in LMICs from market shaping and delivery perspectives.

I confirm that I have read this submission and believe that I have an appropriate level of expertise to confirm that it is of an acceptable scientific standard.

# Peer review: Reviewer response for version 2

**DOI:** 10.21956/gatesopenres.14289.r28582

**Published:** 2020-03-02

**Authors:** Jorge Mestre-Ferrandiz

**Affiliations:** 1 Department of Economics, Universidad Carlos III, Madrid, Spain

This paper by Chalkidou and colleagues is very timely and relevant, providing a nice summary of the different theories underpinning the various forms and types of ‘differential pricing’ to improve access to medicines globally, but in particular in low and middle-income countries (LMICs). The authors also provide a roadmap for next steps, which policy makers should certainly reflect and act upon, in my view. I have advocated before for value-based differential pricing (VBDP) – and while value based tiered pricing (VBTP) as considered by Chalkidou *et al.* has some important nuances on the factors affecting (relative) price levels relative to VBDP (i.e. prices should not be based solely on average income per capita), the same overarching principles apply. Thus, I would support the recommendations offered by this timely paper that “While VBTP is no magic bullet for universal LMIC access to medicines, it holds the potential to move countries forward toward universal health coverage (UHC)”.

 I have three main comments related to three broad issues which the authors could provide further thoughts and insights, given their relation with VBTP:

1. First, authors could discuss in greater detail the use of differential pricing within countries, especially in middle income countries where income inequality is high. I assume the same rationale applies to global VBTP, and where benefits of VBTP could be maximised? I believe there is already some examples of differential pricing across countries (see for instance the work published by Access to Medicine Foundation here and here).
2. The authors discuss nicely some of the pre-requisites and challenges of implementing VBTP, but I miss a discussion of the implications of the WHA Resolution on “transparency of prices”, and in particular whether these moves hinder or favour VBTP. I feel that the implications of this resolution are wider than “just” affecting the feasibility of VBTP; specifically on VBTP, it could potentially hinder its application as it could leave little room for different prices globally.
3. I think one of the most important comments of this paper is: “The debate, therefore, is not about the value of differential pricing per se but rather how differential pricing should be structured; **how it should distribute surplus between the manufacturer and buyer(s);** and, relatedly, how it should trade off between dynamic and static efficiency.” I am aware of other work from the authors discussing how to distribute the surplus, but I was wondering if the authors could provide their view on how this distribution could be agreed?

I have a number of other minor points, which are generally about clarifying some of the comments made by the authors. These are as follows:

 Page 2: “leading to tensions between different global agencies and country governments that further complicate this issue”: this seems a very important barrier/challenge – any recommendations from authors on how to resolve part of these tensions?

 Page 3: “they later recoup their upfront investments **and** earn profits by selling successful pharmaceutical innovations at prices well above marginal cost, protected by term-limited patents”: Agreed with this comment, although it could be possibly argues that companies will recoup their R&D costs IF they earn profits sell from a successful innovation (rather than AND).

 Page 3: “Prices are not determined by the costs of R&D; however, the size of investment in R&D is determined by the expectations of the global prices manufacturers are likely to command for the products in development”. I agree prices should not be determined by R&D costs, although we still have examples of cost-plus price regulation? Also, I would argue the size of investment in R&D is indeed determined by price expectations, but this factor will not be the only one determining R&D expenditure.

 Page 3: “Since post-hoc sales indirectly fund R&D in this way,”: directly, rather than indirectly?

 Page 4: “Even though Ramsey pricing can be welfare efficient, it may not be consistent with the goal of improving access to LMICs”. Why do authors argue Ramsey pricing may not be consistent with this goal?

 Page 4: “Without consensus on what would be the socially acceptable rate of return for the manufacturer, the selected price differentials often transfer a larger portion of surplus to the manufacturer, making it similar to a price discriminating monopolist”: I have two comments with this sentence. First, is there any evidence supporting the point that manufacturers receive a larger proportion of surplus? Second, I would argue that if we could reach a global consensus on VBTP, agreeing an acceptable rate of return would be much easier to do – albeit still complicated!

 Page 4: the reference to Scherer (2004) is about an alternative way of having differential pricing, although not for the same product, but by having multiple products.

 Page 4: ref to Hellerstein *et al.*, (2004): out of curiosity, does this article comment on impact on access to ARVs of not having differential pricing?

 Page 5: “In addition, while overall welfare may be higher, tiered pricing may allow producers to charge a margin which is far higher than the amount needed to recoup R&D investments and production costs (Moon *et al.*, 2011)”. Is this an opinion by Moon *et al*, or do they offer empirical evidence about the level of margins?

 Page 5: “or through competition (via compulsory licensing)”: This phrase could imply that compulsory licensing is the only way to increase competition, but I would argue there could be better ways to promote competition (both therapeutic and generic) other than via compulsory licensing.

 Table 1: this is a nice summary table – I was wondering if we should consider whether the context where treatment is dispensed (primary care pharmacy or hospital) could affect the feasibility of differential pricing?

 Page 6: “Many LMICs are already using or exploring value assessment (through health technology assessment) to inform their procurement/price negotiation and reimbursement decisions,…”: although in the Background section you mention that “The lack of a clear architecture for pricing and prioritization of health products continues to be a major impediment to achieving UHC” and that many health products get funded that should not be funded?

 Figure 1: this is also a nice Figure – one question I have is whether the two options under “Coordination mechanism and/or pooled procurement” are mutually exclusive or complementary?

 Page 7, and references to “Estimates of the marginal productivity of health expenditure”: these estimates are certainly very useful, but I would argue the authors need to also flag some of the critiques around these estimates (e.g. here). Moreover, even though authors provide a reference (Woods *et al*, 2016) to argue that these estimates could be used to give some indication of possible values for lower income countries, it could be seen as an inconsistency with other remarks you make about the important differences across countries. I would welcome the authors own views on this issue.

 Page 7: Paragraph starting “VBTP therefore offers…”: Could VBTP imply higher prices in some countries vs status quo?

 Page 7: “By using the benefits-based price as the starting point/ceiling price in negotiations with industry, country payers can ease budget pressure, making collective or universal coverage more politically and financially sustainable”: I agree with this point, but if VBTP is used to set maximum prices, then further negotiations leading to price discounts would imply that the “net” price will not really reflect “value”?

 Page 9: Section on “Pre-requisites for VBTP”: I miss in this section having separated markets as a pre-requisite; for example, with the current system of international reference pricing, is VBTP feasible?

 Page 9: “The demand curves for individual patients’ out-of-pocket demand are very from population level willingness to pay,”: missing word “DIFFERENT” between “are” and “very” i.e. out-of-pocket demand are very DIFFERENT from population…

 Page 10: Refs Nemzoff *et al.*, 2019 and Syam, 2014: Do the authors think these 2 initiatives merit a bit more information?

 Page 10: “and access to medicines advocates who fiercely (and correctly, in our view) oppose a model of value-based pricing dictated by the healthcare products industry.”: I guess everyone opposes to anything that is dictated by any single agent. We need some kind of overall agreement.

 Page 11: “Assessment of evidence-informed prices and volumes”: list or net prices?

Does the article adequately reference differing views and opinions?

Partly

Are all factual statements correct, and are statements and arguments made adequately supported by citations?

Partly

Is the Open Letter written in accessible language?

Yes

Where applicable, are recommendations and next steps explained clearly for others to follow?

Yes

Is the rationale for the Open Letter provided in sufficient detail?

Yes

Reviewer Expertise:

Economics of life sicences industry

I confirm that I have read this submission and believe that I have an appropriate level of expertise to confirm that it is of an acceptable scientific standard.

# Peer review: Reviewer response for version 1

**DOI:** 10.21956/gatesopenres.14278.r28537

**Published:** 2020-02-10

**Authors:** Hector E. Castro

**Affiliations:** 1 Pharmaceutical Economics & Financing, Management Sciences for Health (MSH), Arlington, VA, USA

The document is relevant and very interesting, and opens an opportunity for global debate. However, it will require some adjustments:

1. "private companies pay upfront for pharmaceutical R&D (though they often benefit from public sector investments in basic scientific research and early stage R&D)” - add a couple of references here (page 2).
2. Maybe add a graph to contrast high, low and potential optimal price? (page 3).
3. "Manufacturers must be able to securely separate economic markets based on demand elasticity" – perhaps it is worth mentioning the effect on demand due to forced displacement of big chunks of the population from one LMIC to a neighboring country affecting elasticity of demand? (page 4).
4. "Many LMICs have highly skewed income distributions, making it more lucrative to cater to richer segments of society than the more voluminous poorer classes" (page 4) - perhaps mention something about the ability of LMICs to aggregate demand on behalf of the population through UHC? Reducing skimming of those able to pay?
  “This is exacerbated by relatively low levels of pooling; most purchase decisions, in practice, are made by individuals" - this applies mostly to LIC, but worth discussing the case of MIC (page 5).
5. "Pooled procurement has been deployed, for example...Pan-American Health Organization (PAHO) Revolving Fund for Vaccines" (page 6) – it is worth adding the Strategic Fund from PAHO for Hep C and other costly drugs.
6. "Under VBTP, prices in each country should be based on a health system’s willingness to pay, where willingness to pay reflects the actual, assessed value of the product within that market/health system (‘value-based pricing’) (See Figure 1) (Claxton, 2007)" (page 6) - is this a realistic assumption with resources available in LMICs? Should be added as a major challenge.
7. "Many LMICs are already using or exploring value assessment (through health technology assessment) to inform their procurement/price negotiation and reimbursement decisions, including Thailand, China, India, Ghana, South Africa, the Philippines, Indonesia, Brazil Mexico, Colombia, Kenya and Tanzania" (page 6) – this is worth clarification, there are many ad-hoc exercises with different levels of success and political buy-in.
8. "Potential challenges (pages 9-11) - What if a global agreement is not possible?":

- Competing agendas of donors vs burden of disease and needs of LMICs?
- Lack of systematic priority setting in many LMICs?
- Starting with NCDs or products affected by aid transition? Or by local burden of disease?
- Who should play the role of the agency? A global body, regional clusters or local bodies to decide and inform on VBTP?
- Limited local capacities to assess value?
- The issue of using thresholds with limited/low quality data or by-passing mechanisms to grant market access affecting elasticity of demand (i.e. judicial intervention)? Inability to say 'no'?
- Lack of trust and political will from payers and manufacturers?
- Two sides of the coin advocates for TRIPS flexibilities vs full access to innovation regardless of cost?
- Other variables affecting ex-factory prices of medicines (e.g., mark ups, taxes)?
- Transition from donations to tier pricing (i.e. commodities for NTDs)?
- The issue of oligopolies under unfair competition/low quality standards (i.e. branded generics)?
- Lack of ability to mobilize more resources for health of many LMICs affecting cash flows? Even if interventions are worth doing?

Does the article adequately reference differing views and opinions?

Partly

Are all factual statements correct, and are statements and arguments made adequately supported by citations?

Partly

Is the Open Letter written in accessible language?

Yes

Where applicable, are recommendations and next steps explained clearly for others to follow?

Partly

Is the rationale for the Open Letter provided in sufficient detail?

Yes

Reviewer Expertise:

Health systems, pharmaceutical policies, health technology assessment, health financing, health economics, market dynamics, health reform

I confirm that I have read this submission and believe that I have an appropriate level of expertise to confirm that it is of an acceptable scientific standard.

# Response

**Published:** 2020-02-19

**Authors:** Lydia Regan

We thank the reviewer for his comments; we have amended the paper to address his points. In brief, we have added more references as requested, explained/qualified various statements and pointed to his review for a list of challenges in making VBTP a reality.
